# An Observability Metric for Underwater Vehicle Localization Using Range Measurements

**DOI:** 10.3390/s131216191

**Published:** 2013-11-27

**Authors:** Filippo Arrichiello, Gianluca Antonelli, Antonio Pedro Aguiar, Antonio Pascoal

**Affiliations:** 1 Dipartimento di Ingegneria Elettrica e dell'Informazione, Università degli Studi di Cassino e del Lazio Meridionale, Via G. Di Biasio 43, 03043 Cassino (FR), Italy; E-Mail: antonelli@unicas.it; 2 Faculty of Engineering, University of Porto (FEUP) Rua Dr. Roberto Frias, s/n 4200-465 Porto, Portugal; E-Mail: pedro@isr.ist.utl.pt; 3 Laboratory of Robotics and Systems in Engineering and Science (LARSyS), IST, University of Lisbon, Av. Rovisco Pais 1, 1049-001 Lisbon, Portugal; E-Mail: antonio@isr.ist.utl.pt

**Keywords:** range-only localization, observability metric, underwater vehicle

## Abstract

The paper addresses observability issues related to the general problem of single and multiple Autonomous Underwater Vehicle (AUV) localization using only range measurements. While an AUV is submerged, localization devices, such as Global Navigation Satellite Systems, are ineffective, due to the attenuation of electromagnetic waves. AUV localization based on dead reckoning techniques and the use of affordable motion sensor units is also not practical, due to divergence caused by sensor bias and drift. For these reasons, localization systems often build on trilateration algorithms that rely on the measurements of the ranges between an AUV and a set of fixed transponders using acoustic devices. Still, such solutions are often expensive, require cumbersome calibration procedures and only allow for AUV localization in an area that is defined by the geometrical arrangement of the transponders. A viable alternative for AUV localization that has recently come to the fore exploits the use of complementary information on the distance from the AUV to a single transponder, together with information provided by on-board resident motion sensors, such as, for example, depth, velocity and acceleration measurements. This concept can be extended to address the problem of relative localization between two AUVs equipped with acoustic sensors for inter-vehicle range measurements. Motivated by these developments, in this paper, we show that both the problems of absolute localization of a single vehicle and the relative localization of multiple vehicles can be treated using the same mathematical framework, and tailoring concepts of observability derived for nonlinear systems, we analyze how the performance in localization depends on the types of motion imparted to the AUVs. For this effect, we propose a well-defined observability metric and validate its usefulness, both in simulation and by carrying out experimental tests with a real marine vehicle during which the performance of an Extended Kalman Filter state observer is shown to depend on the types of motion imparted to the vehicle.

## 1. Introduction

Autonomous Underwater Vehicles (AUVs) have received increasing attention in the last few decades, and nowadays, their application domain spans military, research and commercial operations. AUVs may, for example, be used to support marine biologists in oceanographic environmental monitoring or to execute underwater operations that would be too complex or risky for human operators. To properly execute autonomous operations, the localization of AUVs is of paramount importance. However, as is well known, localization devices, such as Global Navigation Satellite Systems (GNSSs), are ineffective underwater, due to the attenuation of electromagnetic radiation, and they can only be used when the vehicles resurface temporarily. A common approach for AUV localization is to rely on dead reckoning techniques that allow for the estimation of an AUV position by properly merging measurements obtained with inertial and velocity sensors. However, dead reckoning suffers from numerical drift, due to the integration of sensor noise, as well as sensor bias and drift and may be prone to the presence of external currents and model uncertainties; thus, it can only be used for relatively short dives. In practice, this may imply that the AUV will be required to come to the ocean surface periodically to take a new GNSS position fix. Other common solutions for underwater localization are based on the use of acoustic devices that compute ranges between two points and/or determine the bearing and elevation of a point with respect to another using acoustic signals. Among the commercially available solutions, Long, Short and Ultra-Short Baseline systems have found widespread use in challenging applications. In Long Baseline (LBL) acoustic localization systems, a set of transponders is installed at fixed, known underwater positions. While submerged, the AUV computes its distance to each of the transponders by interrogating them and measuring the round-trip travel time of the acoustic waves emitted. The position of the AUV is then computed using a trilateration algorithm. In Short Baseline (SBL) systems, three or more transducers are mounted on a surface vessel, at relative distances on the order of tens of meters. In this case, it is up to the surface segment to request replies from a transponder installed on-board the submerged AUV and to compute its position by triangulation. In Ultra-short Baseline (USBL) systems, a set of transducers is assembled in a compact stand-alone device installed on board a support ship; the estimation of the AUV transponder position is done by measuring the relative phases among the signals arriving at the transducers in response to queries by the surface segment. In the latter two systems, there is a need to transmit back to the AUV its estimated position.

More recently, a different approach based on a single transponder (beacon)/transducer couple has shown good potential to drastically simplify the process of AUV localization while resorting to cost-effective sensors. With this approach, the AUV can position itself by complementing measurements of its range to a single transponder in a fixed known location with complementary information available from other sensor suites installed on board the AUV; namely, depth, inertial velocity and acceleration measurements available from a depth sensor, a Doppler log and an inertial measurement unit, respectively This localization problem, commonly known in the literature as *single beacon localization* or *range-only localization*, has received increasing attention in the very last few years, as pointed out in a number of recently available papers, of which [1–4] are representative examples.

This paper focuses on the single beacon localization problem and proposes an observability metric related to the local weak observability properties for a specific non-linear system. For this reason, and also for the sake of completeness and clarity, in what follows, we give a brief summary of previous relevant work in the literature that addresses the above issues.

In one of the first papers on single beacon localization [1], a Synthetic Long Baseline (SLBL) localization system that uses a single transponder and multiple range measurements taken by an AUV at different locations along its trajectory is presented. The paper describes the key SLBL concepts, proposes an Extended Kalman Filter implementation of the position estimator and discusses SLBL error sources, together with their impact on the localization accuracy. The PhD thesis of Hartsfield [5] gives a summary of the state-of-the-art on single beacon localization. Observability properties are not studied formally, but the concept of unobservable movements is heuristically discussed (e.g., for radial motion).

In [2], an Extended Kalman Filter for single-beacon localization is proposed, and the analysis of the observability conditions for the linear time-varying system obtained by linearizing the nonlinear system about a nominal trajectory is performed. The observability study of planar single beacon navigation for AUVs in the presence of ocean current is presented in the PhD thesis in [6].

In [7], the authors present an observability analysis of the single beacon localization problem, prove local weak observability properties of the underlying non-linear system and discuss the presence of indistinguishable trajectories. In [8], the authors presented an algebraic approach to position estimation for the single-transponder navigation problem.

The authors in [3,9] present a discrete-time localization approach for single beacon localization that, at each sampling instant, enumerates possible solutions to the localization problem and selects the most appropriate by minimizing a proper cost function. A generalization of this approach, complemented with experimental tests with an autonomous surface vessel (equipped with GNSS) and an underwater vehicle, is presented in [10]; the work reported makes ample usage of the Woods Hole Oceanographic Institution (WHOI) acoustic modem [11], configured as a range measuring device. The work in [12] contains an observability analysis for the non-linear system and draws a comparison among different kinds of non-linear filtering techniques.

In [13], the authors analyze the observability of a Global Positioning System/Inertial Navigation System and study the relations among observability, observability measures, estimation error covariance and the information matrix associated with the problem at hand. It is shown that the observability measures adopted have direct connections with the singular value decomposition of a properly defined information matrix. In contrast to what happens with the error covariance, the measures are determined by the system model and are independent of the initial error covariance.

In [14], a solution to the single-beacon navigation problem in the presence of unknown ocean currents is presented; in the proposed approach, at each sampling instant, the relative position of the vehicle with respect to an underwater transponder is firstly computed using a multilateration-based approach, after which a Kalman Filter is used to refine both the position and the current velocity estimates.

Observability issues for the problem of single beacon localization of autonomous vehicles are addressed in [15]. In the case considered, the vehicle is equipped with a standard Inertial Measurement Unit (IMU) to obtain angular velocity readings and with a sensor that measures ranges with respect to a single source. A state augmentation technique is used to derive a linear time-varying system that mimics the dynamics of the nonlinear system, and a study on the observability of both the linear time-varying and non-linear systems is presented. Finally, a Kalman Filter is developed for the Linear Time Varying (LTV) system and tested in numerical simulations.

The results of deep water field experiments on single beacon localization using an Extended Kalman Filter (EKF) are reported in [4]. In the specific case considered, an AUV and a support vessel were equipped with the WHOI acoustic modem; the relative localization algorithm was tested in an area of about 1 km^2^, and an LBL system was used to evaluate the performance of an EKF.

In a recent paper [16], an approach to deal with the distortions effects of the sound-ray propagation in order to get accurate range measurements is presented, and its application to multi-robot underwater relative localization is discussed.

In [17], a state augmentation technique for nonlinear systems to identify observable and unobservable motions for a 3D unicycle-like kinematics model is presented; the proposed approach provides a tool to compute all and only the sets of indistinguishable states in equations for a given set of inputs.

On a different, yet related, vein, the stochastic approach introduced in [18] exploits the concept of a informational correlation coefficient inherited from signal theory to study the degree of observability of a discrete-time, stochastically autonomous system. The Fisher Information Matrix is used in [19] as a tool to obtain binary information about the observability of a nonlinear mapping. The results point to the possibility of using the singular values of the matrix to define a measure of observability. In [20], a bearings-only tracking algorithm is described, and the observability for the non-linear system is discussed applying a rank criterion to the observability matrix. In [21], the problem of finding optimal maneuvers for bearings-only tracking systems is addressed, and a solution based on the Fisher Information Matrix is proposed. In [22], the authors discuss the local weak observability properties for the mobile robot calibration problem and introduce the concept of continuous symmetry to determinate indistinguishable regions, *i.e.*, configuration spaces where all the Lie derivatives are invariant. In [23], a set of observability indexes for the problem of industrial robot kinematic calibration is presented, and analytical properties of the indexes are derived in order to allow for a comparison; among them, the inverse of the condition number of the observability matrix is considered.

An in-depth analysis of controllability and observability properties for non-linear systems is presented in [24]. For important background material, the reader may wish to consult the book [25], while an abridged overview of the main concepts of observability, Lie derivatives and observability rank condition can be found in [22,26] in the context of relative localization.

In [27], two tools, *i.e.*, the local unobservability index and the local estimation condition number, are introduced to measure the degree of observability or unobservability of unforced systems (that is, systems with no inputs). Both measures are defined based on the singular values of the local observability Gramian of the linearized system. In order to simplify the computations, the paper shows how to build an approximate, empirical local observability Gramian. The authors of [28] exploit the use of dynamical optimization and associated computational methods to define and quantitatively measure the observability of a nonlinear system.

Despite the fact that several localization algorithms have been proposed in the literature, a detailed analysis of the observability properties of this specific problem and of its effect on the localization performance are lacking. This circle of ideas is the key motivation for the present paper. In a very general setting, we address observability issues related to the problem of relative localization of two AUVs equipped with velocity and depths sensors, as well as inter-vehicle acoustic ranging devices. The case of single beacon, single AUV localization follows from the general case. Tailoring observability concepts for non-linear systems, we derive a specific observability index (metric) and study its dependence on the types of motion imparted to the vehicles, *i.e.*, we show that the degradation of localization performance depends on the range between the vehicles and the angle between the relative velocity vector and the position vector. Finally, we validate the approach evaluating, both through numerical simulations and experimental tests, the performance of an Extended Kalman Filter state observer as a function of the type of AUV motions.

This paper extends the work reported in [29,30], which built on the results available in [31], and also on the recent paper in [32], which represents a preliminary version of the present work. The extension of [32] is manifold, in that we afford the reader a broad perspective of the state-of-the-art on the topic, include a discussion on observability issues for the problem at hand and carry an in-depth analytic analysis of the observability conditions; moreover, we present the results of numerical simulation studies and an extended series of field experiments.

The paper is organized as follows: In Section 2, we introduce a model for the system under study, investigate its locally weak observability properties and propose a specific observability metric. In Section 3, we present numerical tests to evaluate the performance of an Extended Kalman Filter localization observer in different case studies. Section 4 describes the results of experimental tests with a real marine vehicle localizing a submerged transponder using range measurements from acoustic devices. Finally, in Section 5, we derive some conclusions and discuss issues that warrant further research.

## 2. Observability Study

### 2.1. System Model

**Figure 1.**
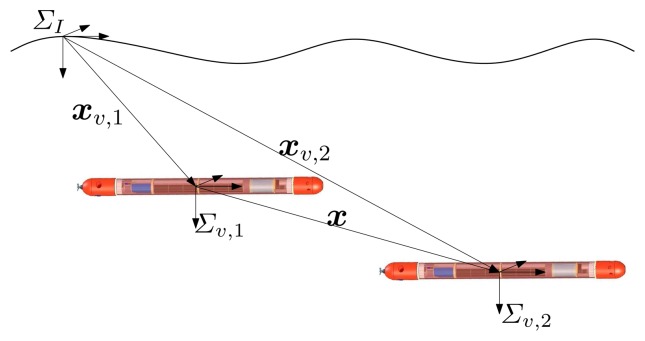
Reference systems: *Σ_I_* is the inertial, earth-fixed frame and *Σ_υ,i_* are the moving frame with the origin fixed in the *i*-th vehicle (*i* = 1, 2).

We start by addressing the problem of relative localization between two AUVs. Let *Σ_I_* be a inertial, earth-fixed, reference frame defined according to the North East Down (NED) convention, and let *Σ_υ,i_* be a frame with its origin fixed in the *i*-th vehicle (*i* = 1, 2) centroid and parallel to *Σ_I_* (see Figure 1). In what follows, ***x**_υ_*,_1_ ∈ ****ℝ****^3^ and ***x**_υ_*_,2_ ∈ **ℝ**^3^ denote the positions of the two vehicles with respect to *Σ_I_*, while ***υ**_υ_*,_1_ = d***x**_υ_*,_1_/d*t* and ***υ**_υ_*,_2_ = d***x**_υ_*,_2_/d*t* denote the inertial velocities of vehicles 1 and 2, respectively. We define the relative velocity between the vehicles as ***υ*** = ***υ**_υ_*,_2_−***υ**_υ_*,_1_ and the system state as ***x*** = ***x**_υ_*,_2_−***x**_υ_*,_1_. We assume it is possible to measure the distance between the vehicles, as well as their inertial velocities and depths. With this notation, a first order kinematic model that captures the relative motion of the vehicles is given by:

(1) $$\{\begin{array}{l} \dot{\text{x}}=\upsilon \\ \text{y}=\left[\begin{array}{c} h_{1}(\text{x})\\ h_{2}(\text{x}) \end{array}\right]=\left[\begin{array}{c} h_{1}\left(∥\text{x}∥\right)\\ {\text{x}}_{3} \end{array}\right] \end{array}$$

where ***x*** is the state, ***υ*** is the input and ***y*** ∈ **ℝ**^2^ is the measurable output vector consisting of a function, *h*_1_, of the Euclidean distance, ‖***x***‖, between the two vehicles and their depth difference, *x*_3_.

In the literature, the range-based localization problem is usually studied in 2D. Here, we start by explicitly including a third dimension (depth) to fully capture maneuvers that include diving. Once we have shown that the vertical component does not effect the observability of the system, we then move on to the 2D case. Notice also that, since the above model is defined in terms of relative motions, it captures the kinematics of both single beacon, as well as relative multi-vehicle localization problems.

To discuss the observability properties of the system in Equation (1), we first recall some basic observability concepts for non-linear systems, summarize important local weak observability properties and describe an observability rank condition; we then apply the latter condition to our specific case.

### 2.2. Observability of Nonlinear Systems

Consider a general class of continuous-time nonlinear dynamic systems described by:

(2) $$\{\begin{array}{ll} \dot{\text{x}}= & \text{f}\left(\text{x},\text{u}\right)\\ \text{y}= & \text{h}\left(\text{x}\right) \end{array}$$

where ***x*** ∈ **ℝ***^n^*, ***u*** ∈ **ℝ***^p^* and ***y*** ∈ **ℝ***^m^* are the state, input, and output vectors, respectively, ***f*** is the state vector field and ***h*** is the output measurement equation. Two initial states, ***x***_1_, ***x***_2_ ∈ **ℝ***^n^*, of the system (2) are said to be indistinguishable if, for every admissible input function, ***u***, the corresponding outputs, ***y***, are identical on their common interval of existence; otherwise, they are distinguishable. Let ***x***_1_, ***x***_2_ ∈ **ℝ***^n^* be two initial states of the system (2), and let *V* be an open set containing ***x***_1_, ***x***_2_. At a local level, ***x***_1_, ***x***_2_ are said to be V-indistinguishable in time *T* if, for every admissible constant input, ***u***, with the property that the states remain in *V* for *t* ≤ *T*, the output functions are the same for *t* ≤ *T*. The system is locally weakly observable at *x*_0_ if there exists an open neighborhood, *U*, of ***x***_0_, such that, for every open neighborhood, *V*, of ***x***_0_ contained in *U*, *I_V_* (***x***_0_) = ***x***_0_, where *I_V_*(***x***_0_) is the set of points V-indistinguishable from ***x***_0_; moreover, the system is locally weakly observable if it is so at every ***x***. Stated simply, local weak observability at ***x***_0_ corresponds to the possibility of finding at least one input, such that ***x***_0_ is distinguishable from all its *close* states, or, at a global level, a system is locally weakly observable if it is possible to instantaneously distinguish each initial state from its neighbors [24,25,33].

Local weak observability can be inferred from the observability rank condition in [24,25] (theorem 3.32, page 95), that yields a *sufficient condition* for it. Before enunciating the latter condition, we first recall the definition of Lie derivatives of the scalar output, *h_j_*, in Equation (2) as:

(3) $$\begin{array}{lll} L_{f}^{0}h_{j} & = & h_{j}\\ L_{f}^{1}h_{j} & = & \nabla h_{j}\cdot \text{f}=\overset{n}{\underset{i=1}{\text{∑}}}\frac{\partial h_{j}}{\partial x_{i}}\cdot f_{i}\\ L_{f}^{2}h_{j} & = & \nabla \left[L_{f}^{1}h_{j}\right]\cdot \text{f}\\ \cdots & & \\ L_{f}^{n}h_{j} & = & \nabla \left[L_{f}^{n-1}h_{j}\right]\cdot \text{f} \end{array}$$

with ∇ denoting the gradient operator and $L_{f}^{\alpha }h_{j}$ being the set of the *α*-order Lie derivatives for any *j* ∈ {1,…, *m*}. The observability rank condition states that the system (2) is *locally weakly observable* at ***x***_1_ if there exists an input, ***u***, such that the matrix:

(4) $$O=\left[\begin{array}{c} \nabla L_{f}^{0}h_{j}\\ \nabla L_{f}^{1}h_{j}\\ \vdots \\ \nabla L_{f}^{k}h_{j} \end{array}\right]$$

computed at ***x***_1_ is full rank for some index, *k* ∈ **ℕ**; in the above, $L_{f}^{\alpha }h_{j}$ is the set of the *α*-order Lie derivatives for any *j* ∈ {1,…, *m*}.

### 2.3. Observability Analysis

Consider the system in Equation (1), where, for convenience in the analytic computation, we define $h_{1}\left(∥\text{x}∥\right)=\frac{1}{2}{\text{x}}^{\text{T}}\text{x}$, that is:

(5) $$\{\begin{array}{lll} \dot{\text{x}} & = & \upsilon \\ \text{y} & = & \left[\begin{array}{c} \frac{1}{2}{\text{x}}^{\text{T}}\text{x}\\ x_{3} \end{array}\right] \end{array}$$

where ***x*** = [*x*_1_ *x*_2_ *x*_3_]^T^ and ***υ*** = [*υ*_1_ *υ*_2_ *υ*_3_]^T^. The observability test yields the matrix:

(6) $$O=\left[\begin{array}{c} \nabla L_{f}^{0}h_{1}\\ \nabla L_{f}^{0}h_{2}\\ \nabla L_{f}^{1}h_{1}\\ \nabla L_{f}^{1}h_{2}\\ \nabla L_{f}^{2}h_{1}\\ \vdots \end{array}\right]=\left[\begin{array}{c} {\text{x}}^{\text{T}}\\ \left[\begin{array}{ccc} 0 & 0 & 1 \end{array}\right]\\ \upsilon^{\text{T}}\\ 0^{\text{T}}\\ 0^{\text{T}}\\ \vdots \end{array}\right]=\left[\begin{array}{ccc} x_{1} & x_{2} & x_{3}\\ 0 & 0 & 1\\ \upsilon_{1} & \upsilon_{2} & \upsilon_{3}\\ 0 & 0 & 0\\ 0 & 0 & 0\\ & \vdots & \end{array}\right]$$

In this case, the rank condition implies that it is possible to select a velocity input that ensures locally weakly observability for all ***x*** ∈ **ℝ**^3^, such that ***x*** ≠ [0 0 ⋆]^T^; that is, for the cases where the vehicles are not aligned along the same vertical axis; in the degenerate case *x*_1_ = *x*_2_ = 0, the rank criterion is not satisfied. The vertical component of the state, as expected, does not influence the observability analysis, because it is measured. It is important to notice that, since the observability rank condition is only a sufficient condition, its failure at a point does not necessarily imply local unobservability [25].

In the following, we want to study the observability problem when the relative velocity input cannot be arbitrarily chosen to satisfy the rank condition. In this case, the system does not have an (actuated) input, and the relative velocity, ***υ***, can be viewed as an exogenous signal to which it is not possible to impose a desired value. From the rank criterion, it is possible to claim that the system is locally weakly observable at any ***x*** under the action of any ***υ***, such that *x*_1_*υ*_2_ ≠ *x*_2_*υ*_1_. The latter condition basically means that the projections of the relative position, ***x***, and velocity vector ***υ*** on the horizontal plane are not parallel, and it admits a simple geometric interpretation: the instantaneous relative direction of motion of the vehicles, given by ***υ***, is not parallel to the vector that joins the two vehicles.

**Figure 2.**
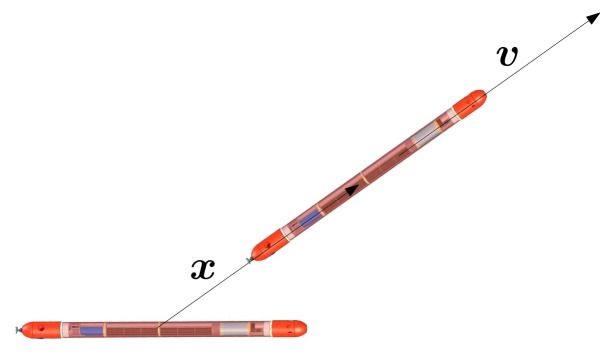
2D-case, where the relative position, ***x***, and velocity, ***υ***, vectors are parallel.

For a given, but otherwise arbitrary, velocity vector, ***υ***, we henceforth call the associated radial domain the set of initial states, such that the projections of ***x*** and ***υ*** are parallel, that is, the set of initial states, ***x***, that do not verify the rank condition for the assigned ***υ*** (see Figure 2). When a state is locally observable, then the rank condition is true in an open and dense subset [25] (theorem 3.35,page 97); if the rank condition is not satisfied in any open set, then the state is not locally observable [27]. Clearly, from these results, no conclusion can be drawn regarding the observability of the states in the radial domain, because the domain itself is not an open set. A direct application of the definition of locally weakly observability, however, allows one to claim that even the states in the radial domain are locally weakly observable with that specific input, since no neighbor of a state belonging to the radial case exhibits its same output. Nevertheless, the radial domain appears to be critical; for example, it may be easily affected by the *kidnapping* problem, not considered in the local analysis [22]. The latter problem is related to the fact that, given a certain input, two initial states, symmetric with respect to the ***x*** vector of the radial domain, despite being locally observable, give the same output and result indistinguishably.

It is important to stress that the observability rank condition provides binary information about observability only and does not give any indication about the degree of observability of a particular system. This observation motivates the next section, where we introduce a metric that can be used to overcome this difficulty.

### 2.4. An Observability Metric for Range-Only Relative and Absolute AUV Localization

So far, we have studied the conditions under which the system described by Equation (2) is locally weakly observable. Notice, however, that the condition obtained, expressed in terms of the rank of the observability matrix in Equation (6), does not provide a measure of the degree of observability of the system. It is for this reason that we now tackle the problem of defining an appropriate observability metric. Since in Equation (6) only three Lie derivatives are different from zero, it seems appropriate to exploit the possibility of building a candidate *observability* matrix, ***O̅***, obtained from 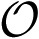 by keeping the three first rows. This yields:

(7) $$\bar{O}=\left[\begin{array}{c} \nabla L_{f}^{0}h_{1}\\ \nabla L_{f}^{0}h_{2}\\ \nabla L_{f}^{1}h_{1} \end{array}\right]=\left[\begin{array}{c} {\text{x}}^{\text{T}}\\ \left[\begin{array}{ccc} 0 & 0 & 1 \end{array}\right]\\ \upsilon^{\text{T}} \end{array}\right]=\left[\begin{array}{ccc} x_{1} & x_{2} & x_{3}\\ 0 & 0 & 1\\ \upsilon_{1} & \upsilon_{2} & \upsilon_{3} \end{array}\right]$$

The proposed metric is related to the numerical conditioning of ***O̅***. It is important to bear in mind that the determinant of a matrix does not give a good measure of how close that matrix is to being singular; instead, the distance of a matrix to the closest singular matrix (as evaluated by the Euclidean-induced matrix norm; is given by its minimum singular value [34] (page 82).

**Figure 3.**
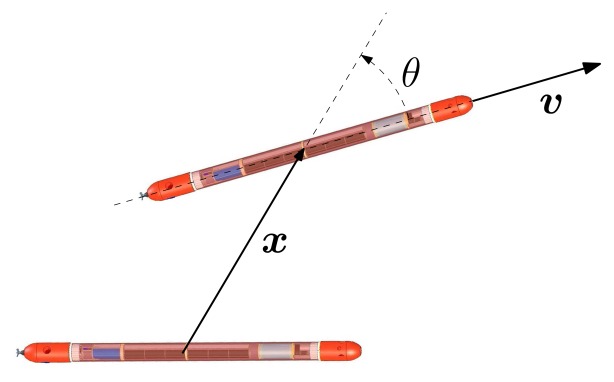
Relevant variables: relative position ***x***, relative velocity ***υ*** and the angle between the vectors, *θ*.

In what follows, without loss of generality, we focus on the model that is obtained from Equation (2) by discarding the vertical motion components and working with the projections of the state and input vectors on the horizontal plane. This is justified by the fact that the vertical components of motion can be measured directly by depth sensors. We therefore address a 2D localization problem. With an obvious abuse of notation (notice how the dimensions of ***x*** and ***υ*** are different in the 3D and 2D cases), the resulting equations can be written as:

(8) $$\{\begin{array}{ll} \dot{\text{x}} & =\upsilon \\ y & =\frac{1}{2}{\text{x}}^{\text{T}}\text{x} \end{array}$$

where ***x***, ***υ*** ∈ **ℝ**^2^ contain the first two components of the vectors defined in Equation (1). The matrix, ***O̅*** ∈ **ℝ**^2×2^, becomes:

(9) $$\bar{O}=\left[\begin{array}{c} {\text{x}}^{\text{T}}\\ \upsilon^{\text{T}} \end{array}\right]$$

and, as expected, its determinant is null when *x*_1_*υ*_2_ = *x*_2_*υ*_1_. To study the numerical characteristics of the matrix, it is convenient to represent the relative position and velocity vectors in polar coordinates as follows:

$$\begin{array}{lll} \text{x} & = & x\;{\left[\begin{array}{cc} \cos\alpha & \sin\alpha \end{array}\right]}^{\text{T}}\\ \upsilon & = & \upsilon \;{\left[\begin{array}{cc} \cos\phi & \sin\phi \end{array}\right]}^{\text{T}} \end{array}$$

where *x* = ‖***x***‖ and *υ* = ‖***υ***‖. Let *θ* = *ϕ* − *α* (see Figure 3). Clearly, the vectors, ***x*** and ***υ***, are parallel when *θ* = 0 or *θ* = *π*. By further defining:

(10) $$\gamma =\frac{x}{\upsilon }$$

it follows that:

(11) $$\bar{O}=\upsilon \;\left[\begin{array}{cc} \gamma \cos\alpha & \gamma \sin\alpha \\ \cos\phi & \sin\phi \end{array}\right]$$

From the above, the condition number, *C* ≥ 1, of ***O̅*** can be computed to yield:

(12) $$C=\frac{\max\left\{\sigma_{1,2}\right\}}{\min\left\{\sigma_{1,2}\right\}}=\frac{\gamma^{2}+1+\sqrt{\gamma^{4}+2\gamma^{2}\cos\left(2\theta \right)+1}}{2\gamma |\sin\left(\theta \right)|}$$

where *σ*_1,2_ are the two singular values of ***O̅***, which, defined as functions of *γ* and *θ*, are given by:

(13) $$\sigma_{1,2}=\upsilon \frac{\sqrt{2}}{2}\sqrt{\gamma^{2}+1\pm \sqrt{\gamma^{4}+2\gamma^{2}\cos\left(2\theta \right)+1}}$$

**Figure 4.**
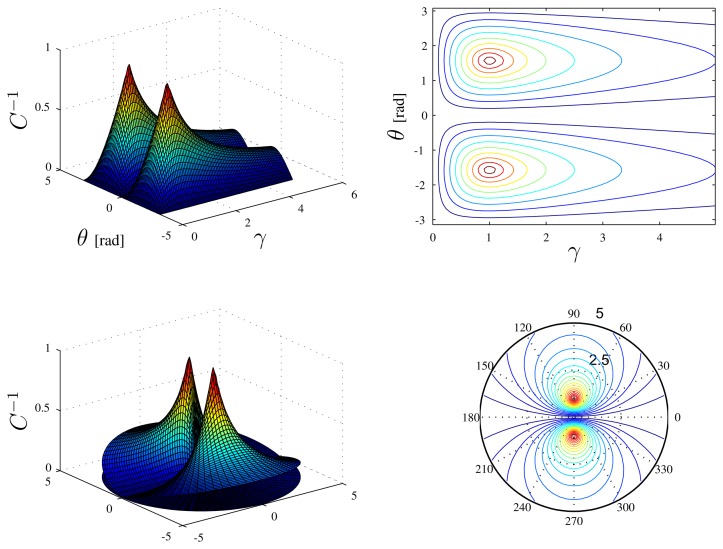
Different views of the inverse of the condition number with respect to *θ* (in radians) and *γ* in Cartesian (top) and Polar (bottom) coordinates. The variable, *θ*, is the angle between relative velocity and position vectors, while the parameter, *γ*, is the ratio between their norms.

As is well known, *C* gives information on how the matrix, ***O̅***, is well conditioned. The inverse of the condition number *C*^−1^, which has a value in the interval [0, 1], is also often used. A zero value indicates that the matrix is singular. Figure 4 shows the inverse of the condition number of *C* as a function of *γ* and *θ* defined above.

It is interesting to notice that *C*^−1^ has a maximum for:

(14) $$\{\begin{array}{ll} \gamma =1 & \left(i.e.,x=\upsilon \right)\\ \theta =\pm \frac{\pi }{2} \end{array}$$

which corresponds to the situation in which the relative position and velocity vectors are orthogonal and have the same magnitude. Notice also that *C*^−1^ is null for *θ* = 0, *θ* = *π*, *γ* = 0 or *γ* → ∞, the latter condition being observed when the ratio between the norms of ***υ*** and ***x*** grows unbounded. The *optimum* condition (maximum of *C*^−1^) can be derived by noticing that, for a fixed *γ*, the determinant of ***O̅*** has a maximum for *θ* = ±*π*/2. This follows from:

(15) $$\begin{array}{cc} \frac{\partial }{\partial \theta }\det\left(\bar{O}\right)=0 & \Rightarrow \gamma \cos\theta =0 \end{array}$$

which corresponds to *θ* = ±*π*/2.

The following table shows how the ratio between *x* and *υ*, defined as the parameter, *γ*, plays an important role as a metric to assess the quality of the observability of Equation (8).

Analyzing the proposed metric, it can be conjectured that, assuming a constant relative speed, the observability conditions and, therefore, the expected performance of any position observer, degrades when the distance between two AUVs (or between an AUV and a transponder in the case of single beacon localization) increases. This fact has been experimentally acknowledged by several researchers (see, e.g., [1]), but has not been formally proven. Notice also that the computation of the index given by the condition number of ***O̅*** requires knowledge of *x*, *υ* and *θ*; while the first two are actually available to the vehicle, the angle, *θ*, cannot be measured/imposed unless the vector, ***x***, between the two vehicles can be measured.

At this point, it must be stressed that the interpretation of the observability conditions allows for a general characterization of the intrinsic limitations that arise in the problem of relative localization. As such, the study is independent of any specific localization algorithm used and affords system designers extremely helpful guidelines. From a practical standpoint, should one wish to actually use the observability index as an aid for on-line motion planning (to steer a vehicles along a trajectory that improves the conditions for good relative localization), then the AUV must necessarily use an estimated value of *θ*. In the singe beacon navigation problem, for example, if the AUV knows an approximate position of the transponder, the on-line planner can compute an approximate value of *θ* and assign an input command to the AUV in a direction that will improve the observability index.

The results derived apply also to the problem of absolute single-beacon localization, which can be viewed as a version of the relative localization problem obtained by making one of the vehicles degenerate into a fixed beacon. In one scenario, the objective is to compute the unknown position of a beacon by measuring the ranges between a moving vehicle (the position of which is known) and the beacon itself. This scenario motivated the experimental tests in Section 4. Another scenario, studied with the help of simulations in Section 3, calls for the computation of the position of a moving vehicle by measuring its ranges to a beacon at a given fixed location.

## 3. Single Beacon Localization: Numerical Simulations

This section presents the results of numerical simulations that illustrate the effectiveness of the observability index proposed in assessing the performance that can be obtained with a single beacon localization algorithm. Specifically, we consider a single-beacon localization algorithm that is obtained by implementing an Extended Kalman Filter for a discrete-time version of Equation (5), and we evaluate its performance under different operational conditions (e.g., different vehicle speeds and trajectories) in light of the proposed metric.

The system state equation is assumed as:

(16) $$\{\begin{array}{c} {\text{x}}_{k+1}={\text{x}}_{k}+T\upsilon_{k}+{\text{w}}_{k}\\ y_{k+1}=\frac{1}{2}{\text{x}}_{k}^{\text{T}}{\text{x}}_{k}+\mu_{k} \end{array}$$

where ***w**_k_* and *μ_k_* are process and measurement noises, assumed as zero-mean Gaussian noise with ***w**_k_* ∼ 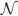 (0, ***R****_w_*) and *μ_k_* ∼ 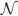 (0, ***R****_μ_*); **T** is the sample time.

An EKF filter for Equation (16) was implemented using the following standard equations:

(1) **Time update of state and estimation error covariance:**
  (17) $$\begin{array}{ccc} {\hat{\text{x}}}_{k+1}^{-} & = & {\hat{\text{x}}}_{k}^{+}+T\upsilon_{k} \end{array}$$
  (18) $$\begin{array}{ccc} {\text{P}}_{k+1}^{-} & = & {\text{P}}_{k}^{+}+{\text{R}}_{w} \end{array}$$
**Measurement updates of state and estimation error covariance:**
  (19) $${\text{K}}_{k+1}={\text{P}}_{k+1}^{-}H_{k}^{T}{\left[H_{k}P_{k+1}^{-}H_{k}^{T}+R_{\mu }\right]}^{-1}$$
  (20) $${\hat{\text{x}}}_{k+1}^{+}={\hat{\text{x}}}_{k+1}^{-}+K_{k+1}\left(y_{k}-\frac{1}{2}{\text{x}}_{k+1}^{-T}x_{k+1}^{-}\right)$$
  (21) $$P_{k+1}^{+}=\left(I-K_{k+1}H_{k}\right)P_{k+1}^{-}$$
  where ***H****_k_* is the linearization of the output measurement equation computed for $\text{x}={\hat{\text{x}}}_{k+1}^{-}$, *i.e.*, ${\text{H}}_{k}={\hat{\text{x}}}_{k+1}^{-T}$.

The covariance matrix of the process and measurement noises are assumed to be ***R****_w_* = *diag*([0.1 0.1]) (where *diag*([*a*_1_ … *a_n_*]) is a diagonal matrix whose diagonal entries starting in the upper left corner are *a*_1_,…, *a_n_*) and ***R****_μ_* = 5, respectively, while the EKF covariance matrix is initialized as ${\text{P}}_{0}^{+}=\text{diag}\left(\left[\begin{array}{cc} 2 & 2 \end{array}\right]\right)$.

**Figure 5.**
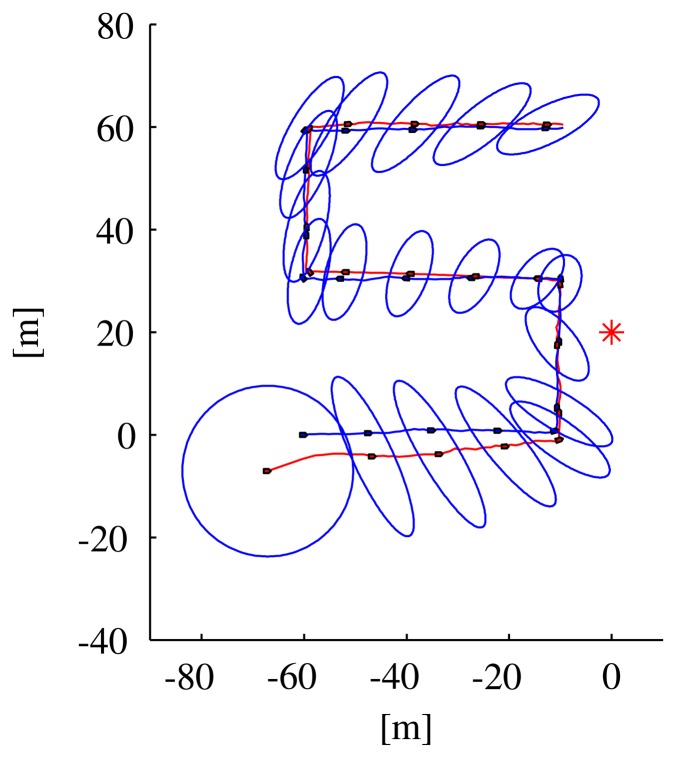
Real (blue) and estimated (red) path of a vehicle moving along orthogonal segments of a line using an Extended Kalman Filter (EKF) and range measurements from a single beacon in position [0, 20] m. The ellipses represent the covariance matrix of the EKF, while the red star represents the position of the transponder.

In the first simulated scenario, the vehicle was commanded to execute a lawn-mowing maneuver (consisting of a succession of orthogonal line segments). The speed of the vehicle was kept constant and equal to 1 m/s along each segment, except when required to change orientation during the transition from one to the next segment; in the latter case, the vehicle was supposed to have the capability to rotate in place. Figure 5 shows the path followed by the vehicle and its position as estimated using the EKF; the figure also shows the ellipsoid corresponding to the EKF covariance matrix along the path.

**Figure 6.**
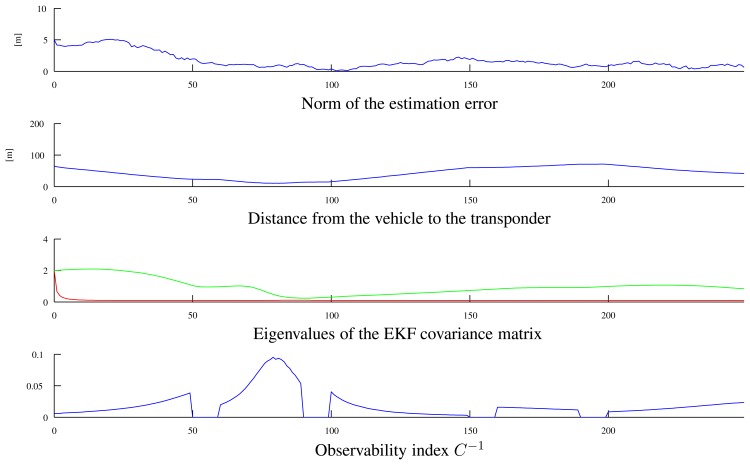
Variables of interest related to the paths in Figure 5: observability index *C*^−1^, eigenvalues of the EKF covariance matrix, distance from the vehicle to the transponder and the norm of the estimation error.

Figure 6 shows the time-evolution of different variables of interest: the norm of the estimation error, the distance of the vehicle from the transponder, the eigenvalues of the EKF covariance matrix and the observability index, *C*^−1^. Notice that the lower eigenvalue of the EKF covariance matrix quickly decreases to a value related to the range measurement covariance; the higher eigenvalue, however, exhibits more complicated dynamics related to the AUV path and decreases when the observability index takes large values. The intervals where the observability index, *C*^−1^, is equal to zero correspond to the rotation of the AUV in place, when it moves from one segment to the following.

**Figure 7.**
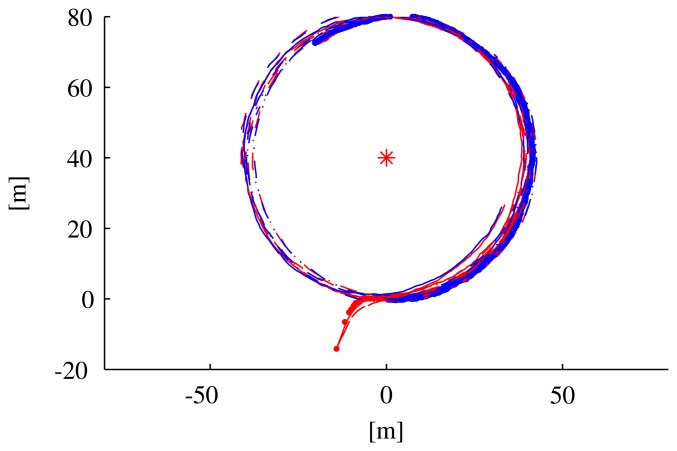
Paths taken by the vehicle along a circumference centered on the beacon, traversed at different speeds (blue), along with the estimated positions (red).

**Figure 8.**
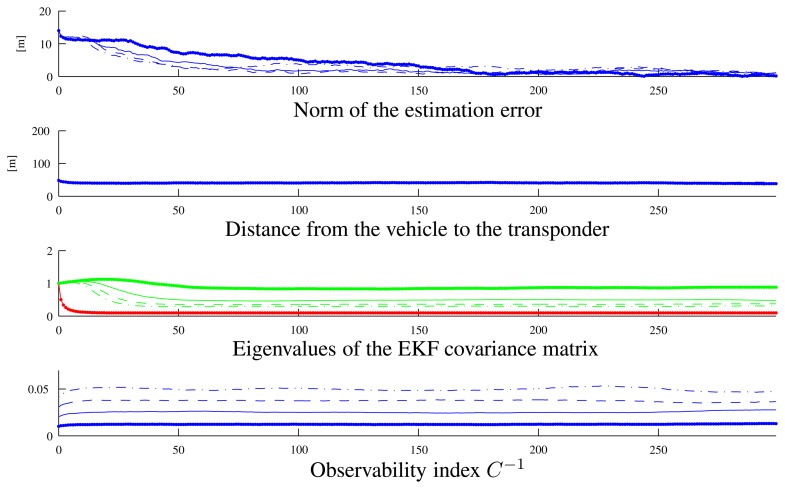
Variables of interest related to the paths in Figure 7: observability index *C*^−1^, eigenvalues of the EKF covariance matrix, distance from the vehicle to the transponder and the norm of the estimation error. Line styles (solid, dotted, dashed) are coherent among the different plots and with Figure 7.

To show a clearer relation between the observability index and the filter performance, we executed two different sets of simulations with the vehicle moving along circumferences centered on the transponder. In the first set of simulations, the vehicle moved along arcs of a circumference of 40 m radius (see the paths in Figure 7) at different velocities (0.5 m/s, 1 m/s, 1.5 m/s, 2m/s), which correspond to different values of the observability index. Figure 8 shows that along the path with a lower instantaneous observability index (solid line, performed at the lowest speed), the estimation error decreases more slowly than in the other cases; identical comments apply to the EKF covariance matrix higher eigenvalues.

**Figure 9.**
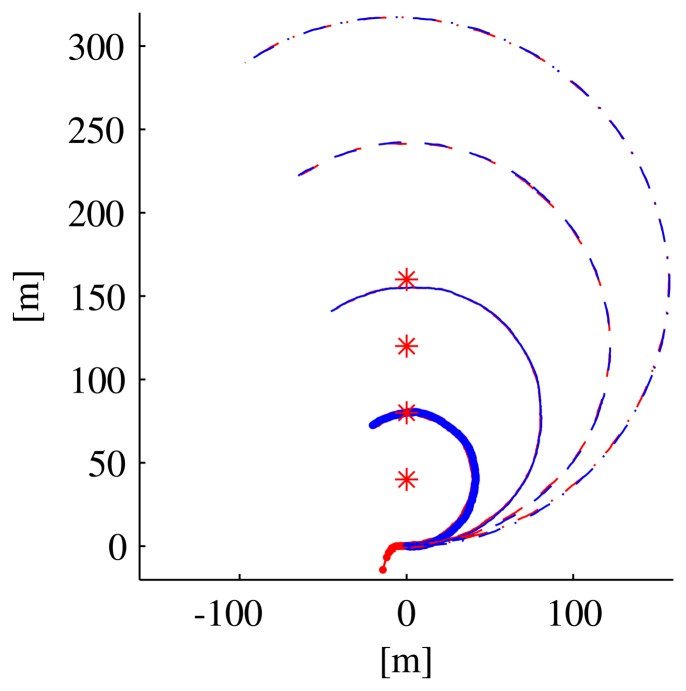
Different circular paths executed by the vehicle at different speeds keeping the same observability index. The transponder is located at the center of each path.

**Figure 10.**
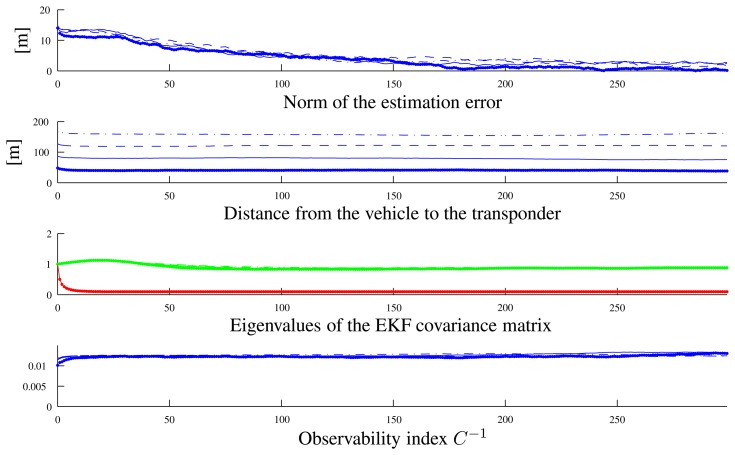
Variables of interest related to the paths in Figure 9: observability index *C*^−1^, eigenvalues of the EKF covariance matrix, distance from the vehicle to the transponder and the norm of the estimation error. Line styles (solid, dotted, dashed) are coherent among the different plots and with Figure 9.

In the second set of simulations, the vehicle was commanded to move along circumferences with different radii at different speeds ((40 m 0.5m/s), (80 m 1 m/s), (120 m 1.5 m/s), (160 m 2m/s)), but keeping the same observability index value. Figure 9 shows the paths of the AUV in the different cases; Figure 10 shows that, despite the different speeds and paths, the same observability index values make the evolution of the EKF estimation errors and covariance very similar.

## 4. Experimental Tests

This section contains the results of practical experiments aimed at assessing the usefulness of the observability index proposed in this paper, in a real scenario. Here, we focus on the closely related problem of beacon detection, *i.e.*, the vehicle knows its position and it must estimate the position of the beacon using range measurements; the latter can be viewed as the dual of the single beacon localization addressed in the previous section. The field tests were executed using an autonomous marine vehicle equipped with an acoustic ranging device capable of measuring its distance to an acoustic transponder fixed at a known position on the seafloor. The tests took place at the Nations Park of Lisbon (the former site of the Expo 98), Portugal (Lat: 38.766, Long: −9.03).

### 4.1. Experimental Set-Up

In the experiments, for simplicity of execution, an Autonomous Surface Vessel (ASV) was used as a proxy for an AUV. This allowed for expeditious testing of the beacon detection algorithm without having to deal with the problem of AUV localization. In fact, with the set-up adopted, the position of the ASV can be measured with a GNSS system. From a practical acoustics viewpoint, however, the difficulty of performing ASV-based beacon detection using range measurements only is comparable to that of AUV-based single beacon localization.

**Figure 11.**
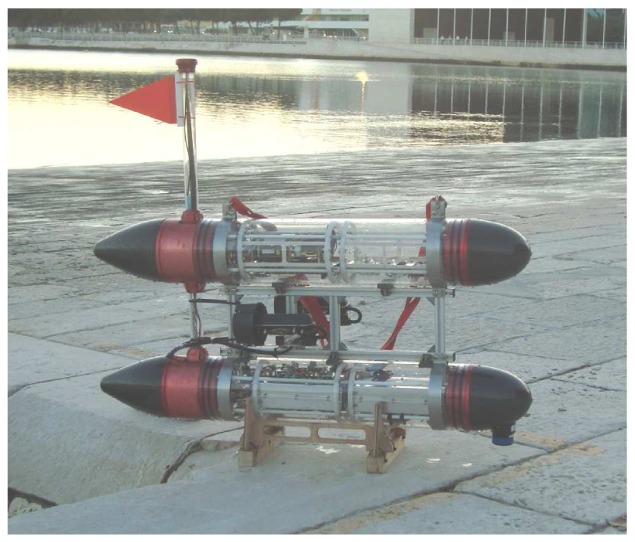
The Medusa autonomous surface vessel.

**Figure 12.**
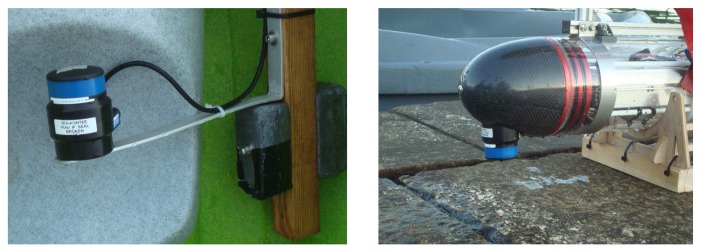
Fixed transponder **(left)** and acoustic modem/ranging device on the Medusa nose cone **(right).**

The ASV used in the experiments, named Medusa (see Figure 11), was developed at the Laboratory of Robotics and Systems in Engineering and Science (LARSyS) of the Instituto Superior Tecnico of Lisbon. The vehicle has two side thrusters that can be independently controlled to yield motions in surge and yaw. The vehicle is equipped with an Attitude and Heading Reference System (AHRS) and a GNSS and can communicate with other devices at the surface using WiFi or with systems underwater, using an acoustic modem that serves also as a ranging unit (Tritech Micron Modem; see Figure 12 (right)). The transponder, installed at a known position underwater at 2 m depth, is essentially a Tritech Modem unit (Figure 12 (left)) configured to respond to queries from the surface modem. Upon receiving a reply, the latter computes the round-trip travel time between the surface and the underwater units and, by using the value of the speed of sound in the water, computes the corresponding distance. For powering and debugging purposes, the transponder was connected via a cable to a surface support system that was properly moored.

Because the computation of the distance between the ASV and the transponder involves the speed of sound in the water, proper care was taken to measure it with a dedicated instrument daily, prior to initiating the tests.

In each of the tests, the ASV was commanded to perform different paths specifically designed for the observability study: a set of parallel/orthogonal segments (some of which were radial with respect to the transponder) or circular paths (centered on the transponder). The ASV data, including GNSS positions, compass readings and range measurements were stored throughout the series of experiments. The data were post-processed to test the performance of an Extended Kalman Filter with analogous equation to those in Equations (17)–(19). The filter was designed so as to be implemented on-board the ASV by taking explicitly into account the low update frequency of the range measurements (one sample every few seconds) and dealing with temporary losses in the range measurements: when no range information is available, the observer is updated by considering only the velocity measurement (as a standard dead-reckoning approach). As in the numerical simulation cases, the covariance matrix of process and measurement noises are assumed to be, respectively, ***R****_w_* = *diag*([0.1 0.1]) and ***R****μ* = 5, while the EKF covariance matrix is initialized as ${\text{P}}_{0}^{+}=\text{diag}\left(\left[\begin{array}{cc} 2 & 2 \end{array}\right]\right)$.

Notice, again, that, from an experimental and even conceptual point of view, the problem of estimating the position of the autonomous vehicle with respect to the transponder is similar to that of estimating the position of the transponder (as if it were unknown) given that the position of the vehicle is known. The tests described here focus on the second scenario.

### 4.2. Experimental Results

Several tests were run, and the data were processed, leading to coherent results among them.

**Figure 13.**
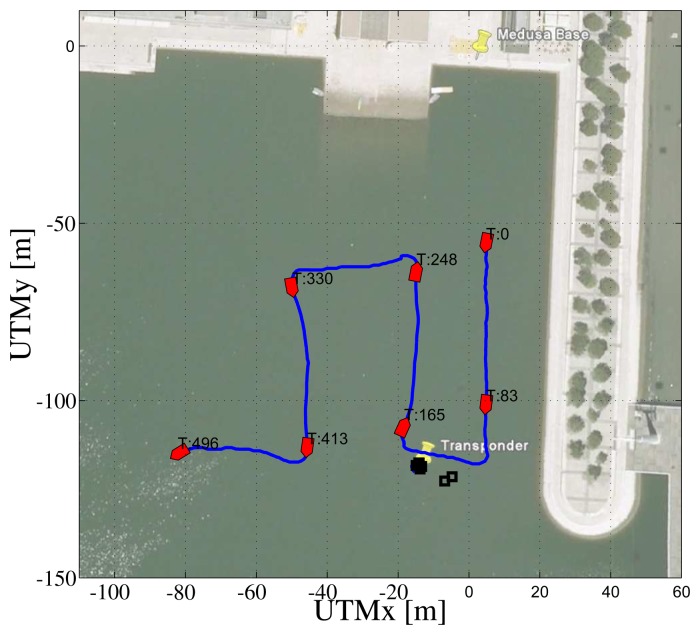
Path followed by the Medusa Autonomous Surface Vessel (ASV) during the first mission.

In the first test, the ASV was commanded to follow a set of parallel and orthogonal segments in an area of about 100 m x 100 m. Figure 13 shows the path of the ASV during the mission. In the background, it is worth noticing the transponder position (marked with the blue star) and the remote base station (Medusa Base) in charge of receiving and storing data from the ASV; the black squares represent the estimated position of the transponder at different times in the course of the mission.

**Figure 14.**
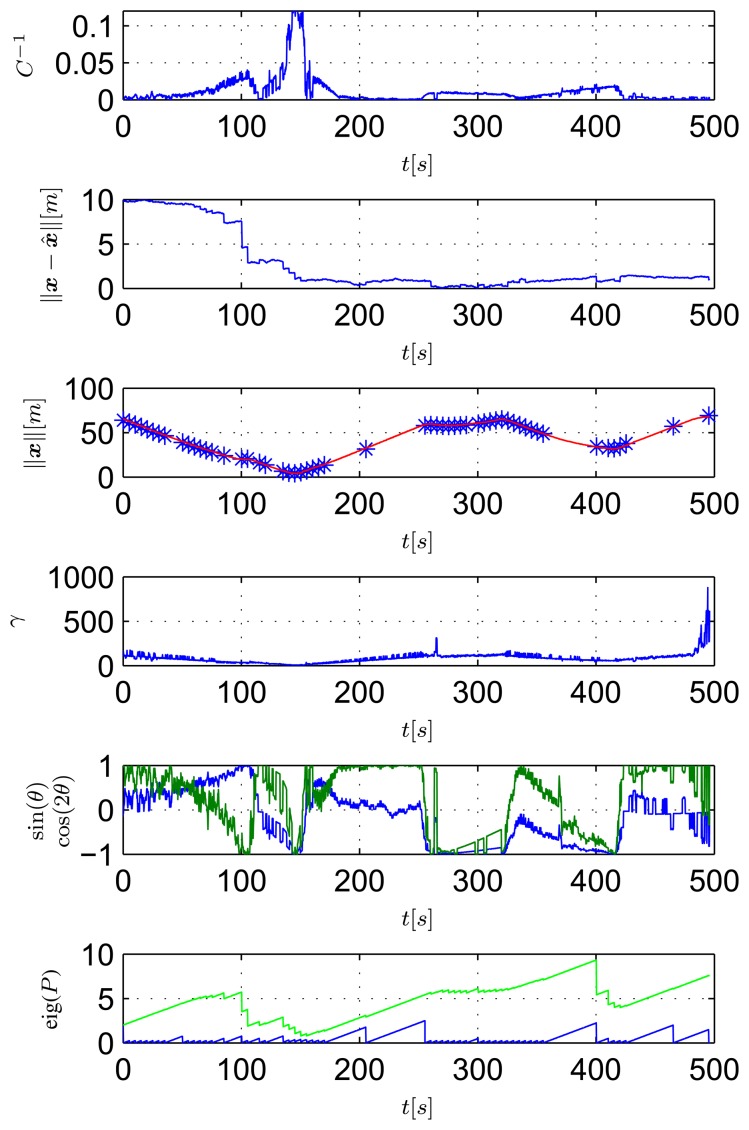
Observability parameters during the first mission: inverse of condition number (top plot), norm of estimation error (second plot from top), measurements of the range between the vehicle and the transponder (blue stars) and GNSS (red line) (third plot), *γ* (fourth plot), sin(*θ*) (blue) and cos(2*θ*) (green) (fifth plot) and eigenvalues of the EKF covariance matrix (last plot).

Figure 14 shows data that are relevant for the observability study The top plot shows the inverse of the condition number of the observability matrix of Equation (9) computed considering real relative displacement. As ground truth data, the real displacement, ***x***, between the transponder and the ASV is obtained using GNSS readings of the ASV and the exact localization of the transponder. The second plot of Figure 14 shows the observer estimation error. The filter is initialized so that the initial estimated position of the transponder is on a circumference centered on the ASV and with a radius equal to the range measurement, at a distance of 10 m from the real transponder position. The third plot of Figure 14 shows the range between the ASV and the transponder measured with the acoustic ranging device (in blue) and also obtained from GNSS data (in red). It is important to notice that the modem measurement is quite accurate; but, the update frequency is slow, and there are some time windows with temporary communication losses (which is expected, in view of the adverse conditions of acoustic propagation in extremely shallow waters). The fourth plots of Figure 14 respectively show the values of γ, sin(*θ*) and cos(2*θ*) (referring to Equation (12)), while the last plots show the higher and lower eigenvalues of the EKF covariance matrix.

At this point, it is important to identify in Figures 13 and 14 some noteworthy mission segments. Notice that when the ASV is moving closer to the transponder (during the 80–180 s time window) the filter error decreases faster than when the vehicle is far from it. Notice also that when no range measurements are available, the filter error is kept low using dead-reckoning data, while both the EKF eigenvalues increase, due to the process measurement noise covariance.

**Figure 15.**
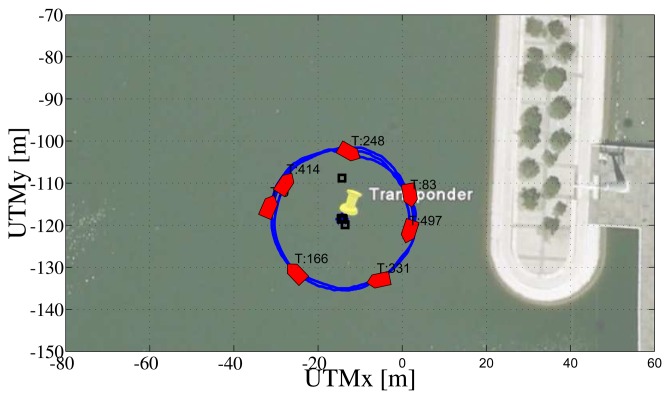
Path of the robot during the second mission.

**Figure 16.**
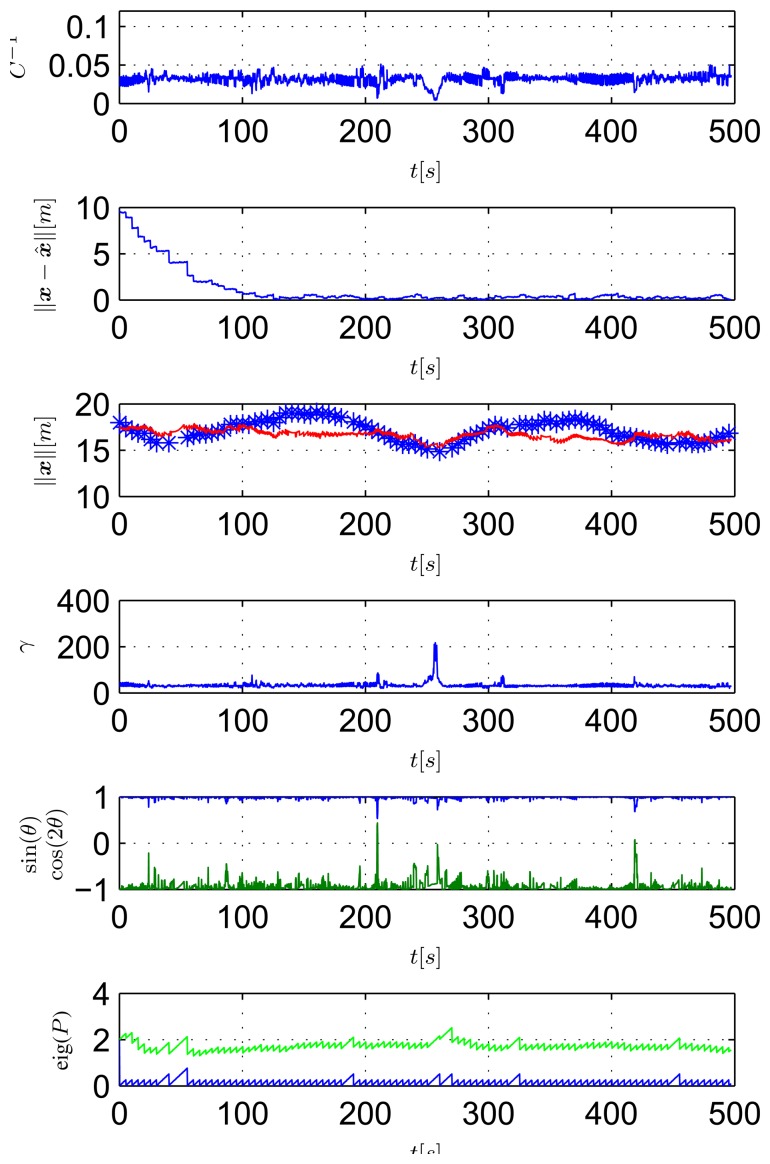
Observability parameters during the second mission: inverse of condition number (top plot), norm of estimation error (second plot from top), measurements of the range between the vehicle and the transponder (blue stars) and GNSS (red line) (third plot), *γ* (fourth plot), sin(*θ*) (blue) and cos(2*θ*) (green) (fifth plot) and eigenvalues of the EKF covariance matrix (last plot).

In the second test, the ASV was commanded to follow a circular path centered on the transponder (see Figure 15). In this case, as seen in Figure 16, the ASV moves with constant *θ* and *γ* (its speed is approximately constant, as well). In particular, $\theta =\frac{\pi }{2}$, so as to maximizes the observability index. Notice that the filter estimation error decreases at a much faster rate and more smoothly than that observed in the first mission; moreover even the higher eigenvalue of the EKF covariance matrix remains bounded during the experiment.

**Figure 17.**
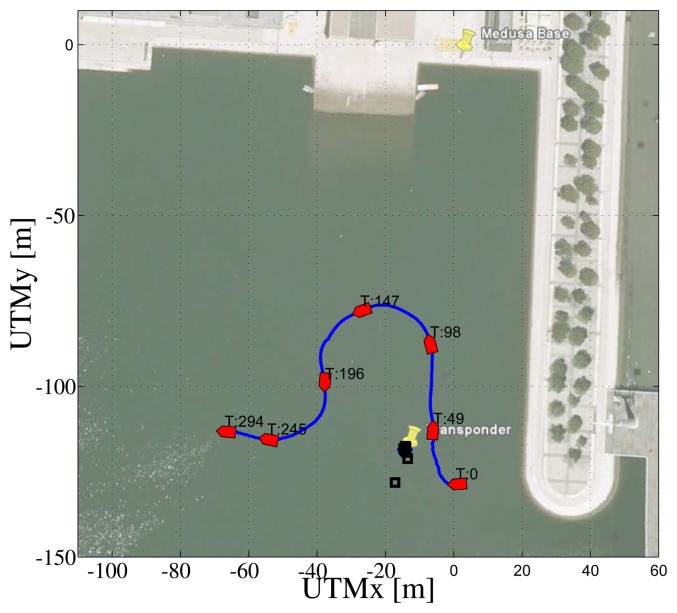
Path of the robot during the third mission.

**Figure 18.**
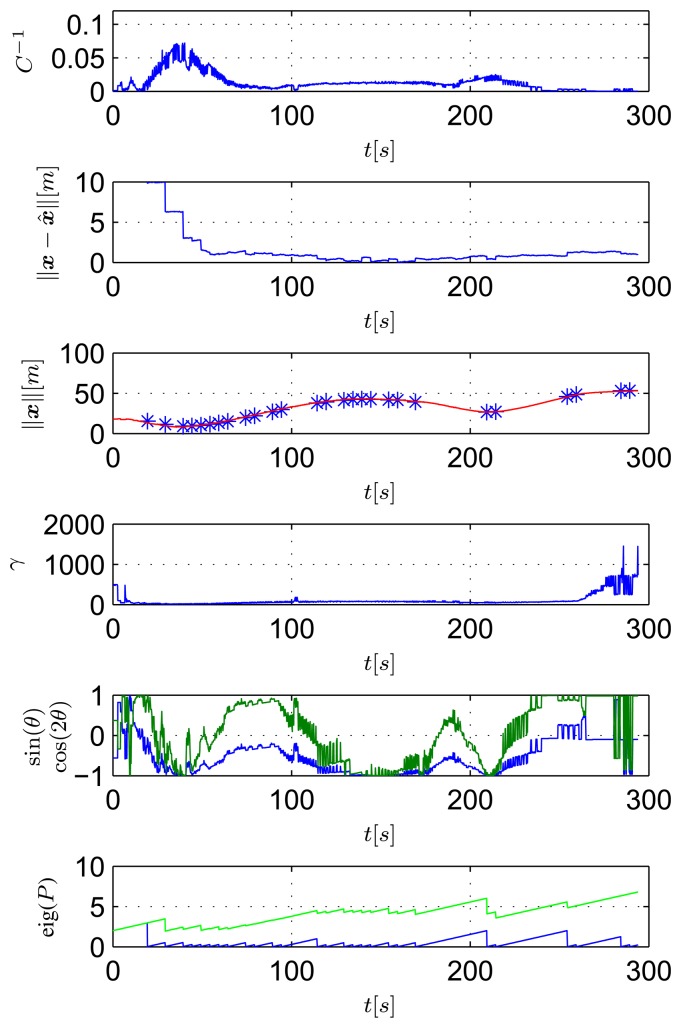
Observability parameters during the third mission: inverse of condition number (top plot), norm of estimation error (second plot from top), measurements of the range between the vehicle and the transponder (blue stars) and GNSS (red line) (third plot), *γ* (fourth plot), sin(*θ*) (blue) and cos(2*θ*) (green) (fifth plot) and eigenvalues of the EKF covariance matrix (last plot).

Finally, the ASV was commanded to follow a sinusoidal path (see Figures 17 and 18); in this case, it is important to notice that, when the observability index is high, the estimation error decreases quickly, and the EKF covariance eigenvalues matrix remains bounded; in the other case, when the observability index is low, the EKF covariance eigenvalues assume higher values. In the final part of the experiment, range measurements are received in a scattered way, and it is possible to notice how the EKF eigenvalues increase due to the AUV process noise covariance.

The multimedia attachment shows a reconstruction from the experimental data of the observability metric analysis relative to the different case studies presented before. The video shows an animation of the ASV motion, the performance of the EKF filter and the dynamic value of the observability index for the different case studies.

## 5. Conclusion

This paper analyzed the structural observability properties of two specific localization problems: single beacon localization and range-only relative position estimation between two AUVs. By exploiting nonlinear observability concepts, a specific metric was proposed to quantify observability with respect to vehicle motions. Simulation analysis and experiments showed the effectiveness of the proposed metric. Future research will address the study of different types of observers, as well as the development of optimal real-time trajectory planning methods that make use of the metric adopted. The extension of this circle of ideas to the multiple vehicle case will also be addressed.

## Figures and Tables

**Table 1.** Minimum singular value and condition number depending on the ratio between *x* and *υ*.

	**Minimum Singular Value**	**Condition Number**
*θ* = 0	0	∞
*θ* = ±*π*/2	min{***x***, *υ*}	$\max\left\{\frac{x}{\upsilon },\frac{\upsilon }{x}\right\}$
